# Serum C1q concentration is associated with disease activity in Chinese Takayasu arteritis patients: A case‐control study

**DOI:** 10.1002/hsr2.252

**Published:** 2021-03-23

**Authors:** Si Chen, Haixia Luan, Jianxun He, Yan Wang, Xiaoli Zeng, Yongzhe Li, Hui Yuan

**Affiliations:** ^1^ Department of Clinical Laboratory Beijing Anzhen Hospital, Capital Medical University Beijing China; ^2^ Department of Clinical Laboratory, Peking Union Medical College Hospital, Peking Union Medical College Chinese Academy of Medical Sciences Beijing China

**Keywords:** C1q, disease activity, Takayasu arteritis

## Abstract

**Background:**

C1q is a crucial component of the classical complement pathway. This study is the first to assess the association between disease activity and serum levels of C1q in Chinese Takayasu arteritis (TA) patients.

**Methods:**

Serum C1q levels in 198 TA patients and 154 healthy controls were assessed, and the relationship between serum C1q levels and indices of TA disease activity was analyzed. Moreover, we examined the correlation between serum C1q levels and two traditional inflammatory biomarkers; erythrocyte sedimentation rate (ESR) and hypersensitive CRP (hs‐CRP).

**Results:**

Serum C1q levels were increased in TA patients compared with healthy controls (*P* = .008). TA patients with active disease had higher levels of serum C1q than patients who had inactive disease (*P* < .0001). In addition, treatment‐naïve patients had higher serum C1q levels than those who had been treated with corticosteroids or at least one immunosuppressant (*P* = .001). Furthermore, a positive correlation between serum C1q levels and traditional inflammatory biomarkers in TA patients was found. The role of C1q in assessing disease activity was studied, and the area under the receiver operating characteristic curve (AUC) of C1q for predicting active disease was 0.752, and a serum cutoff value of 167.15 mg/L C1q maximized the ability of disease activity assessment, with a sensitivity/specificity of 77.80%/64.90%. When the three indicators (C1q, ESR, and hs‐CRP) were combined, the AUC increased to 0.845, and the sensitivity to 84.40%.

**Conclusions:**

The serum C1q is associated with the disease activity of TA and the combination of three indicators (C1q, ESR, and hs‐CRP) increases the sensitivity of disease activity assessment.

## 1 INTRODUCTION

Takayasu arteritis (TA) is an uncommon systemic vasculitis that is characterized by granulomatous inflammation of major blood vessels and primarily involves the aorta and its major branches. Vascular inflammation often leads to ischemia of organs and tissues supplied by the involved vessels and can result in potentially life‐threatening organ ischemia as well as aortic regurgitation and pulselessness.^1^ The active stage of TA is associated with the development of ischemic symptoms such as coronary artery disease, stroke, and vision loss.^2^ Therefore, reliable disease activity assessment is important for preventing TA progression and end‐organ ischemic injury. The erythrocyte sedimentation rate (ESR) and serum C‐reactive protein (CRP) are the biomarkers most commonly used to monitor TA disease activity; however, as they may be influenced by several factors, they are neither sensitive nor specific. In clinical practice, some patients may undergo deterioration of vasculitis without elevation of CRP or ESR, and increases in CRP or ESR are found in only approximately half of patients with active TA.^3^ Consequently, CRP and ESR do not always show a positive association with TA disease activity or severe vasculature impairment.^4^

Complement 1q (C1q) is an important promoter in the classical complement pathway, which is related to the clearance of immune complexes (IC) and apoptotic cells. C1q initiates and activates the complement cascade by recognizing the complement‐binding site of the antibody FC segment in the IgG or IgM immune complex to clear antigen‐antibody complexes.^5^ C1q, which accounts for approximately 70% of the C1 complex, can be deposited on the surface of apoptotic cells, facilitating phagocytosis by phagocytes and protecting the body from the inflammatory reactions.^6^ Interestingly, historical studies demonstrated that C1q could also play a role in other immunoregulatory properties, containing restriction of monocytes differentiation into dendritic cells and the production of immune complex‐induced interferon‐α in plasmacytoid dendritic cells.^7^ Previous studies have found immune complex (IC) in TA patient sera and on peripheral blood lymphocyte Fc receptors.^8^ The pathogenesis of IC in TA is the strong affinity of antigen between aortic wall and complex. Alternatively, antigenic material may be present in the aortic wall.^9^ Serum C1q levels have recently been evaluated in several autoimmune, such as lupus nephritis (LN),^10^ pediatric systemic lupus erythematosus (PSLE),^11^ juvenile idiopathic arthritis (JIA),^12^ and idiopathic inflammatory myopathies (IIMs).^13^ However, the serum C1q level and its association with disease activity have not been investigated in TA patients. This study is the first to examine serum C1q levels in Chinese TA patients and investigate their role in the assessment of TA disease activity.

## 2 MATERIALS AND METHODS

### 2.1 Study population

We designed a retrospective study recruiting 198 subjects with TA diagnosed according to the criteria of the American College of Rheumatology (ACR).^14^ All TA patients were screened in Beijing Anzhen Hospital between September 2015 and August 2019. Patients who had other autoimmune diseases were excluded. A total of 154 healthy unrelated age‐ and sex‐matched controls without any history of chronic disease were recruited during their physical examinations. Disease activity was assessed in patients with TA based on the National Institutes of Health (NIH) criteria proposed by Kerr et al.^15^ Clinical classification of TA (I, IIa, IIb, III, IV, and V) patients was according to the Numano criteria by catheterography or computed tomography angiography (CTA).^16^, ^17^ Written informed consent was obtained from all study participants. The study was approved by the Ethical Committee of Beijing Anzhen Hospital, Capital Medical University.

### 2.2 Measurement of serum C1q level

Serum C1q levels were determined with an automatic biochemical analyzer (AU5400, Beckman) and C1q Reagent Kit (Beijia Biochemistry Reagents Co., Ltd., Shanghai, China). At the same time, blood white cell counts, biochemical parameters, ESR, and hs‐CRP levels were measured. All tests were performed according to the manufacturer's manual.

### 2.3 Statistical analysis

All statistical analyses were conducted with SPSS version 23.0 (SPSS Inc., Chicago, Illinois) software. Numerical data were compared with an independent sample *t* test or the Mann‐Whitney‐Wilcoxon test. Categorical data were compared with the Chi‐square test or Fisher's exact test. Spearman's nonparametric correlation test was applied to examine the associations between serum C1q levels and Kerr's score/ESR/hs‐CRP. We selected the cutoff values for serum C1q, ESR, hs‐CRP, and the combination of three indicators (C1q, ESR, and hs‐CRP) using receiver operating characteristic (ROC) curves with MedCalc software (v.15.2) to compare the accuracies of these markers with disease activity identification. The cutoff points of these markers were the values with the highest Youden's Index (sensitivity + specificity − 1) score. A *P*‐value less than .05 was considered to be statistically significant.

## 3 RESULTS

Of the 198 TA patients, 178 were female, and 47 patients had active disease based on the NIH criteria (Table 1). In addition, 29 of the TA patients in our study were naïve to corticosteroid or immunosuppressant treatment. Malaise (68.2%), headache (46.0%), and chest distress (26.3%) were the three most common constitutional symptoms, and Numano subtype V was common among the TA patients. The prevalence of claudication differed between the TA patients with active and stable disease (*P* = .013). Furthermore, the prevalence of hypertension was notable between untreated TA patients and treated patients (*P* = .002).

**TABLE 1:** Demographic, clinical characteristics, and laboratory findings between TA patients and healthy controls

	HC (Mean ± SEM/*n*)/Median (25%, 75%Q)	TA (Mean ± SEM/n)/Median (25%, 75%Q)	*P*‐value	Active (Mean ± SEM/*n*)/Median (25%, 75%Q)	Inactive (Mean ± SEM/*n*)/Median (25%, 75%Q)	*P*‐value	Untreated (Mean ± SEM/*n*)/Median (25%, 75%Q)	Treated (Mean ± SEM/*n*)/Median (25%, 75%Q)	*P*‐value
Female	136/154	178/198	.634	44/47	134/151	.489	27/29	151/169	.775
Age (years)	38.05 ± 9.07	36.03 ± 12.70	.620	35.79 ± 12.06	36.11 ± 12.94	.972	35.17 ± 12.45	35.47 ± 12.70	.259
Constitutional symptoms									
Fever	/	8/198	/	3/47	5/151	.610	3/29	5/169	.175
Malaise	/	135/198	/	34/47	101/151	.483	19/29	116/169	.739
Arthralgia/Arthritis	/	12/198	/	2/47	10/151	.807	3/29	9/169	.532
Headache	/	91/198	/	20/47	71/151	.592	13/29	78/169	.895
Chest distress/pain	/	52/198	/	14/47	38/151	.530	7/29	45/169	.778
Carotidynia	/	16/198	/	3/47	13/151	.855	1/29	15/169	.534
Vascular findings									
Claudication	/	15/198	/	8/47	7/151	***.013***	5/29	10/169	.080
Bruits	/	127/198	/	30/47	97/151	.959	17/29	110/169	.502
Pulsation weakened	/	143/198	/	34/47	109/151	.983	21/29	122/169	.980
Pulse deficit	/	58/198	/	11/47	47/151	.310	5/29	53/169	.123
Asymmetric BP	/	104/198	/	23/47	81/151	.573	14/29	90/169	.620
Hypertension	/	90/198	/	27/47	63/151	.059	21/29	69/169	***.002***
Laboratory data									
ALT (U/L)	14.40 (9.00‐19.00)	21.39 (11.00‐24.00)	***.002***	19.57 (9.00‐20.00)	21.99 (11.00‐26.00)	.135	22.83 (9.50‐21.50)	21.13 (11.00‐24.50)	.864
Scr (μmol/L)	59.88 (52.50‐68.20)	54.62 (46.75‐61.28)	***<.0001***	54.51 (44.70‐63.30)	54.66 (47.20‐60.70)	.966	54.18 (43.60‐60.50)	54.70 (46.95‐61.50)	.797
WBC (10^9^/L)	5.59 (4.26‐6.30)	7.24 (4.91‐8.40)	***<.0001***	7.40 (5.68‐8.11)	7.19 (4.71‐8.50)	.406	6.05 (4.90‐7.02)	7.46 (4.91‐8.72)	.064
Hb (g/L)	133.99 (125.00‐138.00)	123.30 (114.75‐133.00)	***<.0001***	120.45 ± 14.41	124.24 ± 14.67	.088	124.17 (113.00‐134.50)	123.15 (114.50‐131.50)	.380
PLT (10^9^/L)	257.17 (216.00‐300.00)	238.02 (193.75‐278.25)	***.004***	264.55 ± 80.61	229.30 ± 60.49	***.011***	246.21 ± 78.13	236.55 ± 65.63	.734
ESR (mm/hour)	5.58 (3.00‐8.00)	10.00 (3.00‐12.00)	***.001***	19.66 (10.00‐23.00)	6.80 (2.00‐9.00)	***<.0001***	13.86 (8.00‐15.50)	9.27 (2.00‐11.00)	***.001***
hs‐CRP (mg/L)	0.90 (0.37‐1.03)	3.74 (0.13‐2.29)	.283	9.49 (1.15‐14.88)	1.85 (0.08‐1.17)	***<.0001***	6.40 (0.46‐8.64)	3.26 (0.10‐2.08)	***.001***
C1q (mg/L)	150.11 (143.73‐158.30)	166.85 (140.70‐185.93)	***<.0001***	190.71 (168.10‐209.90)	159.00 (135.60‐178.30)	***<.0001***	187.51 (164.20‐212.45)	163.12 (136.70‐181.65)	***.001***
Numano subtypes									
I	/	9/198	/	3/47	6/151	.771	1/29	8/169	.759
IIa	/	4/198	/	4/47	0/151	***.003***	0/29	4/169	.528
IIb	/	36/198	/	6/47	30/151	.270	2/29	34/169	.088
III	/	7/198	/	3/47	4/151	.448	2/29	5/169	.605
IV	/	17/198	/	3/47	14/151	.750	2/29	15/169	.726
V	/	117/198	/	28/47	89/151	.938	22/29	95/169	.074

*Note*: Bold and italics mean p‐value < 0.05. The reference range of female ESR is 0‐20 mm/hour, and the reference range of male ESR is 0‐15 mm/hour; the reference range of hs‐CRP is 0‐5 mg/L.

Abbreviations: ALT, alanine aminotransferase; BP, blood pressure; ESR, erythrocyte sedimentation rate; Hb, hemoglobulin; HC, healthy controls; hs‐CRP, hypersensitive C‐reactive protein; PLT, platelet; Scr, serum creatinine; TA, Takayasu arteritis; WBC, white blood cell.

**FIGURE 1:**
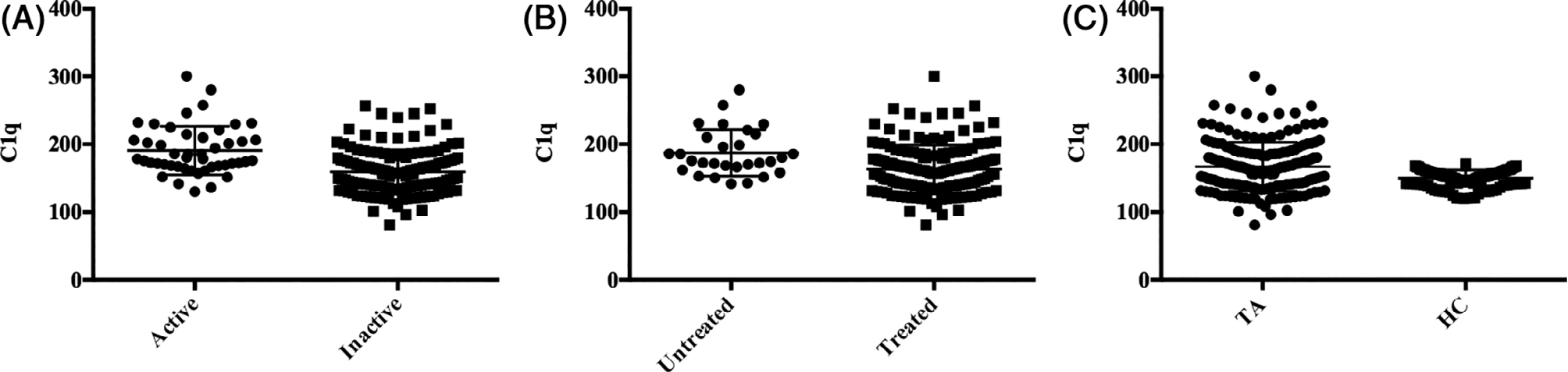
A, Serum C1q levels were significantly higher in TA patients than in healthy controls (*P* = .008); B, Serum C1q levels were significantly higher in TA patients with active disease than inpatients who had inactive disease (*P* < .0001); C, Serum C1q levels were significantly higher in treatment‐naïve patients than in those who had always been treated with corticosteroids or at least one immunosuppressant (*P* = .001). TA, Takayasu arteritis

Compared with the healthy controls, patients with TA had higher ESR and C1q levels (Table 1 and Figure 1). However, the level of hs‐CRP was similar between the TA patients and healthy controls. Compared with patients who had the inactive disease, those with active disease had higher levels of serum C1q and hs‐CRP as well as ESR (Table 1 and Figure 1). Similarly, treatment‐naïve patients had higher serumC1q, ESR, and hs‐CRP than those who had always been treated with corticosteroids or at least one immunosuppressant (Table 1 and Figure 1). We further analyzed the relationship between serum C1qand Kerr's score/ESR/hs‐CRP with the Spearman correlation test and found that in our TA patients, serum C1q levels correlated significantly with Kerr's score, ESR, and hs‐CRP (Table 2).

**TABLE 2:** Correlation of C1q with disease activity in patients with TA

	Kerr's score		hs‐CRP		ESR	
	*r*	*P*	*r*	*P*	*r*	*P*
C1q	0.460	<.0001	0.591	<.0001	0.604	<.0001

Abbreviations: ESR, erythrocyte sedimentation rate; hs‐CRP, hypersensitive C‐reactive protein; TA, Takayasu arteritis.

**FIGURE 2:**
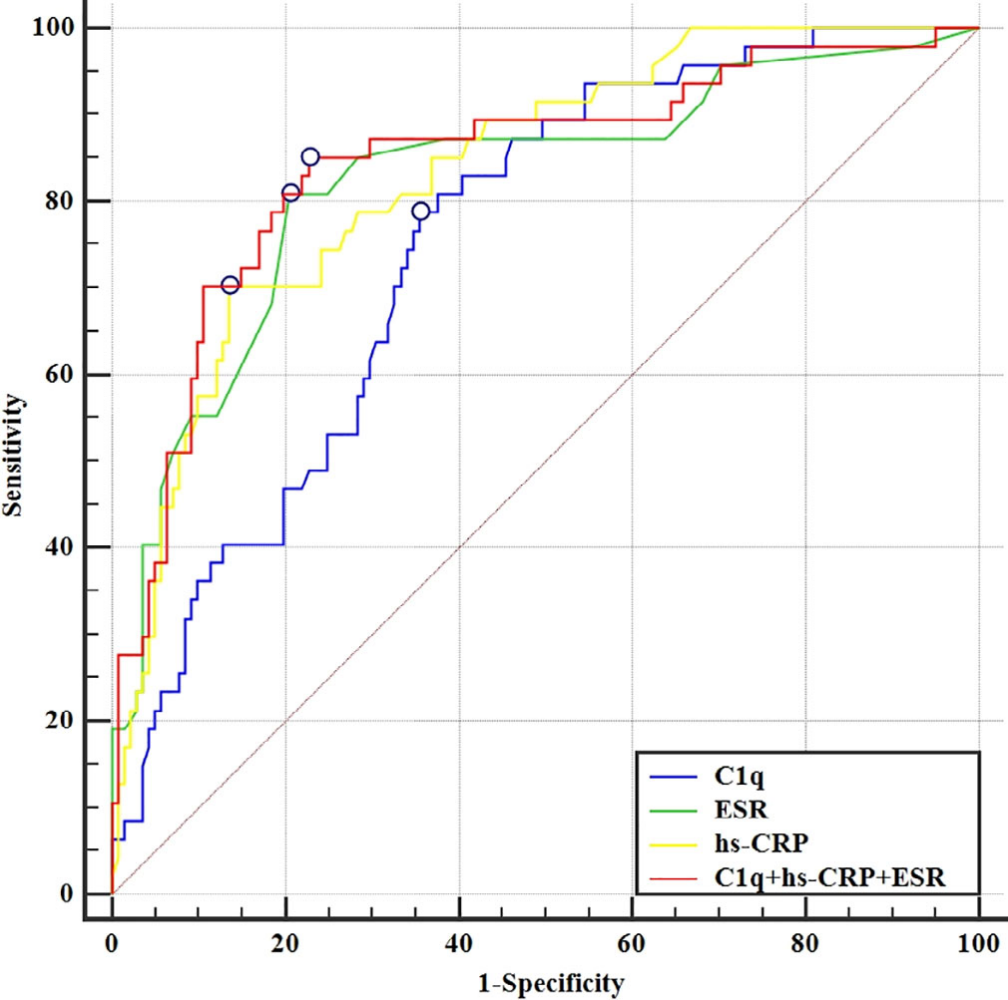
The ROC curves for Cq, ESR, hs‐CRP, and the combination of the three indicators in 190 TA patients according to NIH criteria. AUC, area under the ROC curve; ESR, erythrocyte sedimentation rate; hs‐CRP, hypersensitivity C‐reactive protein; NIH, National Institutes of Health; ROC, receiver operating characteristics; TA, Takayasu arteritis

The areas under the ROC curve (AUCs) for C1q, ESR, and hs‐CRP were 0.752, 0.825, and 0.834, respectively, though without significant differences (Table 3 and Figure 2). Nevertheless, when the three indicators (C1q, ESR, and hs‐CRP) were combined, the AUC increased to 0.845. At the same time, the AUCs between the combination of the three indicators (C1q, ESR, and hs‐CRP) and hs‐CRP with C1q were significantly different from each other. A serum cutoff value of 167.15 mg/L C1q maximized the disease activity assessment capacity, with a sensitivity/specificity of 77.80%/64.90%. Using the established ESR and hs‐CRP thresholds of our center, ESR and hs‐CRP were able to identify disease activity with a sensitivity/specificity of 80.00%/81.70% and 70.20%/86.50%, respectively, and the sensitivity increased to 85.10% when the three indicators (C1q, ESR, and hs‐CRP) were combined (Table 3 and Figure 2).

**TABLE 3:** The sensitivity and specificity of serum C1q and other indicators to assess TA disease activity

	AUC	Sensitivity (%)	Specificity (%)	95% CI	*P*
C1q	0.752	77.8	64.9	0.677‐0.828	<.0001
hs‐CRP	0.834	70.2	86.5	0.770‐0.898	<.0001
ESR	0.825	80.0	81.7	0.748‐0.901	<.0001
C1q + hs‐CRP + ESR	0.845	85.1	77.3	0.775‐0.915	<.0001

Abbreviations: AUC, area under the ROC curve; CI, confidence interval; ESR, erythrocyte sedimentation rate; hs‐CRP, hypersensitive C‐reactive protein; TA, Takayasu arteritis.

## 4 DISCUSSION

This study first analyzed the association of serum C1q levels with disease activity in a large sample of Chinese TA patients. Our experimental results showed that serum C1q levels were increased in TA patients compared to healthy controls. Notably, TA patients with active disease had higher levels of serum C1q than patients who had inactive disease. Serum C1q levels and traditional inflammatory biomarkers in TA patients correlated positively; therefore, our findings show that serum C1q concentrations are associated with the disease activity of TA. TA is a systemic autoimmune vasculitis with an unknown etiology. Immune mechanisms appear to be involved in TA pathogenesis, as inflammatory cell infiltration along with granulomatous inflammation and excessive proinflammatory cytokine productions are observed in the vascular tissue involved.^18^ The pathogenesis of systemic vasculitides, including TA, correlates with the presence of inflammatory cells in the arteries and with IC deposition, which causes activation of the complement system, followed by the inflammation and destruction of vascular mural structures.^19^ Previous studies have found ICs in TA patient sera and on peripheral blood lymphocyte Fc receptors. In addition, a recent paper analyzed the proteomics of circulating ICs in the sera of TA patients and identified three unique antigens.^20^ Although there are some studies on the relationship between IC and TA, analyses of the association between the complement system and TA have not been reported to date. Moreover, whether activation of the complement system is involved in the pathogenesis of TA remains unclear. C1q, historically viewed as the initiating component of the classical complement pathway, has a diverse range of functions in both innate and acquired immunity. Indeed, C1q plays important roles in the pathogenesis of autoimmune diseases, particularly systemic lupus erythematosus (SLE).^21^ However, the relationship between C1q and TA remains unresolved. This study is the first to investigate the serum C1q level in Chinese TA patients and its role in the assessment of TA disease activity.

Several studies have suggested the use of serum C1q as an inflammatory marker in multiple diseases. For example, Tan et al found that the serum C1q level was markedly reduced in LN patients compared with normal controls, revealing a correlation with LN disease activity and renal total activity index scores.^10^ In addition, Wu et al showed that the level of serum C1q in PSLE patients was significantly lower than that in healthy children as well as in children with other rheumatic diseases and that the level of serum C1q correlated negatively with the disease active index.^11^ Furthermore, Gilliam et al reported that in JIA patients, mean serum levels of C1q were significantly increased compared with those in healthy children,^12^ and Li et al indicated significant increases in serum C1q levels in a PM/DM patient group with elevated ESR compared with a group with normal ESR.^13^ Our study found that TA patients with active disease had higher levels of serum C1q than patients who had inactive disease. At the same time, treatment‐naïve patients had higher serum C1q levels than those who had always been treated with corticosteroids or at least one immunosuppressant. We found serum C1q levels to correlate significantly with Kerr's score, ESR, and hs‐CRP levels in our TA patients. The AUC of C1qwas lower than that of ESR, but the AUC of C1q was higher than that of hs‐CRP. In addition, when the three indicators (C1q, ESR, and hs‐CRP) were combined, the AUC increased, and a serum cutoff value of 167.15 mg/LC1q maximized the disease activity assessment capacity. The sensitivity of C1q was lower than that of ESR and higher than that of hs‐CRP, though the specificity of C1q was lower than that of both ESR and hs‐CRP. In addition, when the three indicators (C1q, ESR, and hs‐CRP) were combined, the sensitivity increased. Therefore, hs‐CRP has the highest specificity, and the sensitivity of combining three indicators (C1q, ESR, and hs‐CRP) was the highest. Based on the above, we conclude that the concentration of serum C1qisa potential inflammatory marker for TA and that the combination of the above three indicators increases the sensitivity of disease activity assessment.

There are several limitations to our study. We did not detect C1q deposited in vascular tissue. In addition, using the Kerr criteria to assess disease activity might have missed some TA patients with angiographic activity. As a result, well‐designed prospective studies should be performed to clarify the exact clinical molecular mechanisms of C1q in TA patients and the association with TA disease activity. Also, the clinical application of C1q in TA needs to be expanded.

## 5 CONCLUSION

The present study is the first to assess serum C1q levels in TA patients and evaluate their relationship with disease activity. We found that serum C1q levels were increased in TA patients compared to healthy controls and that TA patients with active disease had higher levels of serum C1q than patients who had inactive disease. In addition, there were positive correlations between serum C1q levels and traditional inflammatory biomarkers in TA patients. Therefore, our findings show that the serum C1q concentration is associated with the disease activity of TA and that the combination of three indicators (C1q, ESR, and hs‐CRP) increases the sensitivity of disease activity assessment, but the exact mechanism of C1q remains unclear.

## CONFLICT OF INTEREST

The authors declare no conflicts of interest.

## AUTHOR CONTRIBUTIONS

Conceptualization: Si Chen, Xiaoli Zeng, Yongzhe Li, Hui Yuan

Data curation: Si Chen, Yan Wang

Formal analysis: Si Chen, Haixia Luan, Jianxun He, Yan Wang

Funding acquisition: Si Chen, Yongzhe Li, Hui Yuan

Investigation: Si Chen, Haixia Luan, Yan Wang

Methodology: Haixia Luan, Xiaoli Zeng

Project administration: Jianxun He, Yan Wang

Resources: Jianxun He, Yan Wang, Yongzhe Li

Supervision: Xiaoli Zeng, Hui Yuan

Writing ‐ original draft preparation: Si Chen, Haixia Luan

Writing ‐ review and editing: Si Chen, Haixia Luan

 All authors have read and approved the final version of the manuscript.

 Hui Yuan had full access to all of the data in this study and takes complete responsibility for the integrity of the data and the accuracy of the data analysis.

## TRANSPARENCY STATEMENT

The lead author affirms that this manuscript is an honest, accurate, and transparent account of the study being reported; that no important aspects of the study have been omitted; and that any discrepancies from the study as planned (and, if relevant, registered) have been explained.

## Data Availability

The authors confirm that the data supporting the findings of this study are available within the article supplementary materials.
